# Lived Experience of Volunteers During Humanitarian Surgical Missions: A Qualitative Study Protocol

**DOI:** 10.3390/nursrep15120435

**Published:** 2025-12-08

**Authors:** Simone Amato, Vincenza Giordano, Alessio Lo Cascio, Daniele Napolitano, Francesco Gravante, Noemi Giannetta, Mauro Parozzi, Mattia Bozzetti, Paola Arcadi, Mariachiara Figura

**Affiliations:** 1 San Camillo Forlanini Hospital, 00144 Rome, Italy; simoneamato7482@libero.it; 2 Department of Public Health, Federico II University, 80131 Naples, Italy; enza-giordano@hotmail.it; 3 Department of Nursing Research and Management, La Maddalena Cancer Center, 90146 Palermo, Italy; 4 CEMAD, Fondazione Policlinico Gemelli IRCCS, 00168 Rome, Italy; 5 Intensive Care Unit, Department of Critical Care, Local Health Authority of Caserta, 81100 Caserta, Italy; 6 Departmental Faculty of Medicine, UniCamillus-Saint Camillus International University of Health and Medical Sciences, 00131 Rome, Italy; 7 Department of Medicine and Surgery, University of Parma, 43126 Parma, Italy; 8 Direction of Health Profession, ASST Cremona, 26100 Cremona, Italy; 9 Bachelor School of Nursing, Università degli Studi di Milano, ASST Melegnano e Martesana, 20063, Milan, Italy; 10 Department of Maternal and Child Health Promotion, Internal Medicine & Specialties of Excellence “G. D’Alessandro” (PROMISE), 90127 Palermo, Italy

**Keywords:** humanitarian mission, volunteers, cleft lip and palate, qualitative research, IPA, protocol

## Abstract

**Background**: Humanitarian surgical missions play a critical role in addressing health disparities, particularly in low-resource settings where conditions such as cleft lip and palate (CL/P) are prevalent. The success of these missions relies heavily on the commitment of volunteers, including healthcare professionals and logistical personnel. While their contributions are widely acknowledged, the psychological and emotional impact of participating in such missions remains underexplored. **Objective**: This study protocol aims to explore the lived experience of volunteers involved in humanitarian surgical missions. **Materials and Methods**: A qualitative study, using Interpretative Phenomenological Analysis (IPA), will be conducted. Semi-structured interviews will be carried out with volunteers who have participated in at least one humanitarian surgical mission. Interviews will be audio-recorded, transcribed verbatim, and analyzed to identify emerging themes. Data collection will continue until saturation is reached. The reporting of this study will follow the COREQ guidelines. **Expected Results**: This study is expected to provide a deeper understanding of the emotional and professional experiences of volunteers in surgical missions. Expected results include identifying key themes related to motivation and preparation, on-site engagement, field experience, interpersonal relationships and group dynamics, and personal reflections. These results will inform strategies to enhance the effectiveness of missions, improve volunteer support, and ensure the sustainability of humanitarian interventions. Additionally, these findings will contribute to the broader field of international health volunteering and support future program development. **Conclusions**: This protocol outlines a rigorous qualitative approach to investigating the lived experience of volunteers in humanitarian surgical missions. The anticipated findings are expected to inform targeted training, psychological support, and organizational strategies, ultimately improving the effectiveness and sustainability of future missions and the well-being of volunteers.

## 1. Introduction

Humanitarian surgical missions represent a key strategy to address global health disparities, particularly in low- and middle-income countries, where access to safe and timely surgical care remains critically limited. It is estimated that nearly five billion people worldwide lack access to affordable surgical care, with children among the most underserved populations [1,2,3]. One of the most frequently treated conditions during these missions is cleft lip and/or palate (CL/P), a congenital anomaly affecting approximately 1 in 700 live births globally, with higher incidence rates reported in African and Asian regions [4,5]. Beyond its physical manifestations, CL/P often leads to nutritional challenges, communication difficulties, and profound psychosocial distress, exacerbated by stigma and cultural beliefs [6,7,8,9]. In this context, several Italian humanitarian initiatives have developed multidisciplinary care pathways to provide comprehensive and sustainable CL/P treatment in underserved areas [10,11,12,13]. These missions mobilize diverse healthcare teams, including surgeons, anesthesiologists, nurses, speech therapists, and logistical volunteers, who collaboratively deliver clinical care alongside capacity-building programs [14,15,16,17,18,19,20,21,22]. The effectiveness of these efforts depends largely on the motivation and commitment of volunteers, who offer their expertise with a strong sense of solidarity to serve populations living in extremely vulnerable conditions [23]. Through coordinated teamwork, these missions respond not only to the medical needs of patients but also to the organizational complexities of humanitarian work. The professionalism and dedication of the teams are essential for ensuring the mission’s long-term impact and sustainability [24].

While previous research has focused primarily on the clinical outcomes of such missions, there is a lack of scientific evidence regarding the subjective experiences of volunteers involved in humanitarian initiatives, particularly in cleft lip and palate surgical missions. Evidence suggests that international health volunteering may foster emotional satisfaction, a sense of belonging, and professional development [25,26,27,28,29]. At the same time, several challenges persist, including emotional exhaustion, role ambiguity, inadequate preparation, and cultural disorientation [12,27]. These experiences unfold in emotionally intense environments marked by urgency, limited resources, and cultural tensions, particularly acute in pediatric care. Volunteers often encounter ethical dilemmas, logistical difficulties, and the emotional burden of witnessing human suffering [26,28,29].

Despite these realities, little is known about how volunteers make sense of their experiences, manage emotional demands, and integrate these missions into their personal and professional identity. To address this gap, this study adopts an interpretative phenomenological approach to explore the lived experiences of volunteers engaged in humanitarian surgical missions. By investigating their motivations, expectations, emotional responses, interpersonal interactions, and post-mission reflections, this research seeks to inform future training, psychological support, and organizational strategies. Ultimately, it aims to enhance the effectiveness and sustainability of humanitarian efforts while shedding light on the emotional, human, and professional dimensions of global health engagement.

### Aim

This study aims to explore the lived experience of volunteers involved in surgical humanitarian missions conducted in low- and middle-income countries, with a specific focus on non-emergency cleft lip and palate surgical missions.

## 2. Materials and Methods

### 2.1. Design

A qualitative study, using Interpretative Phenomenological Analysis (IPA), will be employed [28]. This approach was chosen due to its suitability for an in-depth understanding of participants’ perceptions and experiences, while maintaining a practical and applied focus, especially in complex and poorly explored phenomena. This protocol adheres to the COREQ (Consolidated Criteria for Reporting Qualitative Research) guidelines to ensure methodological transparency and rigor [30].

Interviews will be audio-recorded and subsequently transcribed verbatim for qualitative analysis. Basic sociodemographic information will also be collected.

### 2.2. Setting and Recruitment

Participants will be recruited through direct contact with humanitarian organizations, institutional mailing lists, and, if necessary, snowball sampling. The recruitment period is expected to last approximately 3–4 months, depending on the availability of suitable volunteers within the collaborating humanitarian organizations. Interviews will be scheduled at mutually convenient dates and will be conducted after informed consent has been obtained.

Exclusion criteria will include: (1) volunteers under the age of 18; (2) individuals who have never participated in a humanitarian mission; and (3) participants who are unable to provide informed consent or participate in an interview conducted in Italian.

Interviews will be conducted during the planned data collection period, which is expected to take place between February 2026 and May 2026. Each interview is estimated to last approximately 30–45 min.

A purposive sampling strategy will be adopted to ensure maximum variation in terms of professional role, gender, and number of missions completed. Snowball sampling will be used as a complementary strategy to identify additional participants with relevant experience who may not be easily accessible through formal channels.

To minimize selection bias, all participants will be recruited based on predefined eligibility criteria, and interviews will be conducted solely by researchers with no prior personal or professional relationship with the volunteers.

Recruitment will be conducted by trained research staff. To be eligible, participants must be adults, either healthcare or logistical volunteers, who have participated in at least one humanitarian mission. All participants will receive detailed information about the study, including its aims, procedures, and confidentiality safeguards.

Written informed consent will be obtained voluntarily from each participant before the start of the interviews. The recruitment process will ensure that participants clearly understand the purpose of the study and the nature of their involvement. The final sample size will be determined according to the principle of data saturation, that is, the point at which no new relevant information emerges from additional interviews [31,32].

Sampling and interviewing will continue until saturation is reached, defined as the point at which no new relevant information emerges and existing themes become fully developed and redundant. In qualitative research, this point often referred to as “data saturation” or “thematic saturation” [31] is considered a key indicator of rigor [33]. This study adopts the “code meaning” saturation approach, which assesses saturation not by counting codes but by evaluating the completeness and depth of their meaning. After identifying initial codes, subsequent interviews will be examined to determine whether new aspects or nuances arise; when no further insights are generated, saturation will be considered achieved [31].

### 2.3. Data Collection

Data will be collected through individual, in-depth semi-structured interviews conducted in the Italian language, either in person or remotely via secure online platforms such as Zoom (Zoom Video Communications, v.6.0, San Jose, CA, USA) or Google Meet (Google LLC, v.2024.10, Mountain View, CA, USA). Each interview is expected to last approximately 30–45 min. All interviews will be audio-recorded with the participants’ informed consent to allow for accurate verbatim transcription. For participants who prefer to express themselves in a language other than Italian, a professional translator will support the interview, and translated transcripts will be carefully cross-checked to ensure accuracy and fidelity to participants’ narratives. To ensure semantic consistency, all transcripts will undergo a two-step verification: (1) comparison between the audio recordings and their verbatim transcripts, and (2) comparison between the original-language transcript and its translated version, when applicable. Any discrepancies will be resolved collaboratively by the translator and a member of the research team to preserve participants’ intended meanings. The interview guide will be used flexibly, allowing the interviewer to follow the participant’s narrative without necessarily covering all prompts. A formal pre-interview debriefing is not required, as organizational practices vary; the interview is conceived as a qualitative exploration rather than a debriefing process.

A semi-structured interview guide (Table 1) will be used to explore key dimensions of the volunteers’ lived experience. The guide was developed based on a preliminary review of the literature on international volunteering, humanitarian healthcare work, and psychological outcomes, and was further refined through expert discussion within the research team. The questions address areas such as motivation, expectations, emotional experiences, interpersonal relationships, cross-cultural challenges, and personal or professional transformation.

To ensure clarity and appropriateness, the interview guide will be piloted with one or two eligible participants. Data emerging from the pilot phase will be reviewed by the research team to make any necessary adjustments to the wording, sequencing, or structure of the questions, without including these pilot interviews in the final analysis.

Each interview will be conducted by trained qualitative researchers in a private and comfortable setting to ensure that participants feel at ease.

The IPA approach ensures an emic perspective by prioritizing participants’ subjective meanings and lived experiences throughout the interpretative process. The interview guide is designed to encourage volunteers to articulate their personal interpretations, ensuring that their voices remain central during data collection and analysis.

In addition to the interviews, participants will be asked to complete a socio-demographic questionnaire to provide contextual information. Participation is entirely voluntary, and no financial compensation will be offered.

To ensure independence between researchers and participants, the interviews will be conducted by researchers who have no prior personal or professional relationship with the volunteers involved in the study.

Anonymity will be preserved throughout the entire research process. Each participant will be assigned an alphanumeric identification code, and all potentially identifying information (e.g., names, locations, organizational affiliations) will be removed from the transcripts. Audio files, transcripts, and consent forms will be stored separately on encrypted, password-protected devices accessible only to the research team. In reporting the findings, anonymized quotations identified through alphanumeric codes will be used to prevent any possible identification of participants.

### 2.4. Data Analysis

Participants will be assigned an alphanumeric identification code to protect their anonymity. All interviews will be transcribed verbatim and anonymized before analysis.

Data analysis will be conducted according to the IPA principles outlined by Smith et al. [28]. In the initial phase, each researcher will immerse themselves in the original data. Two researchers will independently begin the analysis by thoroughly examining one transcript at a time. This process involves reading and re-reading the transcripts, making exploratory notes in the margins, developing a summary list of these notes, identifying emerging themes, and exploring connections between them. In cases where disagreements arise, the two researchers will return to the original transcripts and their annotations to reformulate and agree upon shared themes.

After completing the idiographic analysis of each individual interview, the process will be extended to the entire dataset to construct a comprehensive list of thematic summaries, develop clustered thematic groupings, record transcripts with overarching themes, and finalize the theme list supported by illustrative excerpts. This will enable the identification of cross-case patterns while preserving the uniqueness of individual accounts.

The analytic process will follow the core IPA stages: immersive reading, exploratory annotation, development of emerging themes, linking themes, identification of cross-case patterns, and narrative synthesis. Throughout the interpretative phase, attention will be paid to semantic, linguistic, and emotional content to capture the depth and complexity of participants’ lived experiences.

Data analysis will be conducted manually. An audit trail will be maintained throughout the process to ensure transparency and traceability of analytic decisions. In addition, researchers will keep reflective notes and analytic memos to support reflexivity and minimize interpretive bias. Coherence, credibility, and methodological rigor will be ensured through researcher triangulation and constant reference back to the raw data.

Coding will be conducted manually by two independent researchers. No qualitative software will be used. All transcripts will be fully anonymized using alphanumeric identifiers, and data will be stored on encrypted, password-protected devices accessible only to the research team.

The socio-demographic questionnaires will be analyzed with descriptive statistical analysis and calculation of frequencies, percentages, mean, and standard deviation.

### 2.5. Rigor

To ensure the rigor and trustworthiness of the study, we will apply the four criteria proposed by Lincoln and Guba (1985) [34]: credibility, dependability, confirmability, and transferability.

Credibility will be supported by achieving data saturation, by involving multiple researchers in independent and collaborative analysis, and by maintaining methodological consistency throughout the study. Investigator triangulation will allow for multiple interpretations to be compared and integrated, reducing the risk of individual bias.

Confirmability will be pursued using an audit trail, documenting all analytic decisions and changes made during the research process. In addition, researchers will keep reflective memos to critically examine their assumptions and positionality, in alignment with the principles of reflexivity adopted in IPA.

To enhance researcher reflexivity and minimize subjectivity, all members of the research team will maintain reflective memos throughout the study, documenting assumptions, preconceptions, and interpretive decisions. Researcher triangulation will further support the credibility and confirmability of the analytic process by comparing multiple interpretations and returning to the raw data when needed

To enhance dependability, detailed documentation of the research design, interview procedures, and analytic steps will be maintained, ensuring transparency and replicability of the process.

Transferability will be supported by providing a rich description of the study context, participant characteristics, and illustrative quotes from the interviews. The inclusion of socio-demographic data will further support readers in assessing the applicability of the findings to other contexts [35].

Finally, participants will be invited to review a summary of the preliminary findings (member checking) to assess their resonance with the lived experiences described during the interviews.

## 3. Ethical Considerations

The study received ethical approval from the Territorial Ethics Committee Lazio Area 4 (Approval No. 37-2025). All procedures will adhere to the principles of Good Clinical Practice (ICH Harmonised Tripartite Guidelines 1996), the Declaration of Helsinki, and current national regulations on research involving human participants.

Before participation, all volunteers will receive written and verbal information about the study objectives, procedures, risks, and their rights. Written informed consent will be obtained prior to data collection. Participants will be informed that they may withdraw at any time without providing a reason and without consequences.

Data will be processed in accordance with the General Data Protection Regulation (GDPR 2016/679). Audio recordings, transcripts, and personal data will be anonymized through the use of alphanumeric codes. Identifiable information will be stored separately from the research data on encrypted, password-protected devices accessible only to the research team. All documents will be stored securely and retained according to current regulatory requirements.

The study process was designed and implemented to protect the dignity, autonomy, and privacy of participants, in accordance with the principles of beneficence, non-maleficence, and justice recommended for qualitative research in healthcare settings [36,37].

Given that interviews may touch upon emotionally sensitive topics, procedures will be in place to support participants. Interviewers will be trained to recognize signs of distress and will pause or stop the interview if needed. Participants will be offered the possibility to speak with a trained professional or be referred to appropriate support services if emotional discomfort arises.

## 4. Discussion

This study aims to conduct an in-depth exploration of the lived experiences of healthcare professionals volunteering in reconstructive surgery missions in low-income countries.

The expected findings will contribute to strengthening the limited scientific evidence on international surgical volunteering and offer insights for improving the planning, organization, and training of missions in resource-limited settings.

A clearer understanding of volunteers’ motivations, challenges, and emotional responses may support the development of targeted preparation and psychological support strategies, fostering enhanced intercultural collaboration and emotional readiness. These results can guide organizations and policymakers in designing more sustainable and ethically consistent missions.

The study also highlights the relevance of nursing competencies in global and intercultural contexts, promoting the integration of training on international cooperation, stress management, and cross-cultural communication within nursing and allied health education.

Finally, the expected findings may inform practical actions such as tailored training programs, structured psychological support for volunteers, and the promotion of an organizational culture that prioritizes the well-being of healthcare professionals.

## 5. Conclusions

This study will provide new and original insights into the lived experiences of volunteers engaged in surgical humanitarian missions, revealing the emotional, interpersonal, and professional dimensions of global health engagement. By adopting a phenomenological lens, the research will shed light on how volunteers navigate the complexities of intercultural care, ethical dilemmas, and resource-limited environments. The anticipated findings are expected to contribute to the development of targeted strategies that promote emotional preparedness, intercultural competence, and sustainable involvement in global surgery initiatives.

In addition to expanding the scarce literature in this field, the study aims to inform the design of more structured and supportive training pathways, enhance organizational awareness of volunteers’ psychosocial needs, and guide policies that recognize and value human capital in international cooperation. Ultimately, the results may help reframe healthcare volunteering as a transformative professional experience and a key dimension of socially responsive practice.

## Figures and Tables

**Table 1:** Matrix of interview.

Can you share your experience as a volunteer? What motivated you to undertake this activity? How did you prepare yourself for your mission? What were your expectations of the mission before leaving? What were the main challenges you encountered during your fieldwork? Were there any cultural difficulties in the context in which you operated? How was the collaboration with the local and international team during the mission? Can you share a particularly positive moment you experienced during your missions? Can you describe a particularly negative moment you experienced during your missions? What emotions do you feel when you think back to the children and families you helped? How has your professional or personal approach change after returning from the mission? What do you think could be improved to make these missions more effective or sustainable over time? Would you like to repeat a similar experience in the future? Why?

## Data Availability

No data are presented in this manuscript, as it describes a study protocol. The data generated in the future study will be available from the corresponding author upon reasonable request, subject to privacy restrictions.

## Public Involvement Statement

No public involvement in any aspect of this protocol.

## Guidelines and Standards Statement

This manuscript was drafted against the COREQ (Consolidated criteria for reporting qualitative research) for qualitative research.
